# Bilateral Elastofibrolipoma: Distinguishing from Elastofibroma with Adipose Tissue Migration

**DOI:** 10.1155/2015/967670

**Published:** 2015-01-06

**Authors:** Betül Ünal, Ali Uzar, Murat Şedele, Bekir Erol

**Affiliations:** ^1^Department of Pathology, School of Medicine, Akdeniz University, 07058 Antalya, Turkey; ^2^Department of Thoracic Surgery, Antalya Training and Research Hospital, 07058 Antalya, Turkey; ^3^Department of Pathology, Antalya Training and Research Hospital, 07058 Antalya, Turkey; ^4^Department of Radiology, Antalya Training and Research Hospital, 07058 Antalya, Turkey

## Abstract

We present a case of a 54-year-old female patient. MRI examination showed a mass adjacent to the left scapula and a localized heterogeneous mass in the right subscapular area. Microscopic examination revealed abnormal elastic fibers and globules and mature adipose tissue mixed with collagen bands in all areas of the lesion. Genetic analysis was done and there were no changes in DNA copy number. The lesion was diagnosed as elastofibrolipoma which is a rare tumor. No bilateral elastofibrolipoma has been previously described.

## 1. Introduction

Elastofibrolipoma was first defined in 1995 by de Nictolis et al. in the anterior mediastinum [1] and in 2005 by Erkiliç et al. in the subscapular area [2]. It was described as an encapsulated mass of abnormal elastic fibers and connective tissue mixed with mature adipose tissue. de Nictolis et al. classified this lesion as a lipoma variant of a benign neoplasm [1], while Erkiliç et al. dealt with the lesion based on the findings in their case [2]. No bilateral elastofibrolipoma has been previously described. In this paper, a well-defined, bilateral, subscapular lesion in a thin fibrous capsule, consisting of elastic fibers, globules, and bands of connective tissue mixed with fat tissue, was discussed with the relevant literature.

## 2. Case Presentation

**Figure 1:**
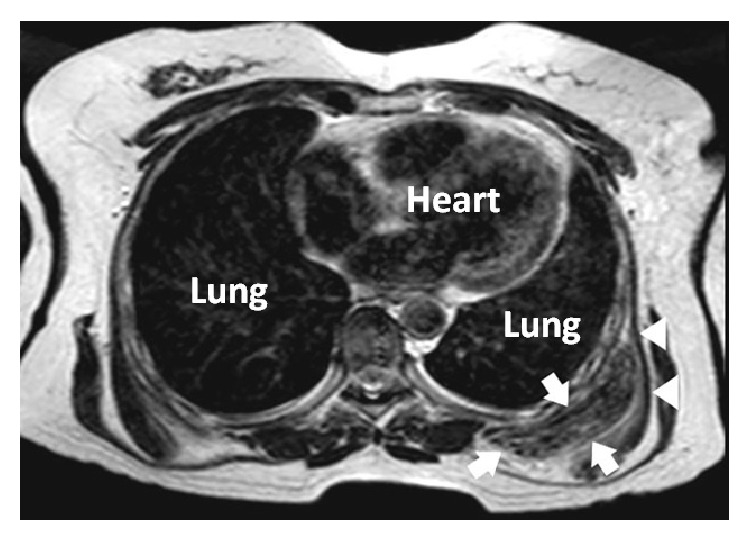
The T1 and T2 weighted MRI examination of the mass showed a localized heterogeneous mass of 64 × 16 mm adjacent to the left scapula. Please note the hyperintense adipose tissue in the form of lines and hypointense fibrous tissue.

A 54-year-old female patient, who increasingly suffered from back pain for 4-5 years, was admitted to our hospital. Physical examination revealed a localized, mobile mass of 10 × 7 cm on the back, in the left subscapular area. The T1 and T2 weighted MRI examination of the mass showed a localized heterogeneous mass of 2.9 × 11 mm with hypointense and hyperintense foci in the right subscapular area (Figure 1(a)) and a mass of 64 × 16 mm adjacent to the left scapula (Figure 1(b)). Subsequently, the larger mass on the left side and the small one on the right were excised.

Macroscopic examination of the mass on the left revealed an encapsulated mass of 9.5 × 9.5 × 5 cm with regular borders, yellow-brown in color, consisting of mature adipose tissue of yellow color, centrally and peripherally, with a flesh-colored elastic consistency among the solid areas. The small mass on the right was encapsulated, measured 2.6 × 1 × 1 cm with irregular borders, and the cut surface was similar.

**Figure 2:**
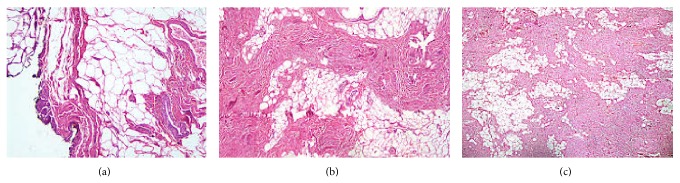
Thin fibrous capsule (H&E, ×200) (a) and a large number of eosinophilic globules, elastic fibers, and collagen mixed tapes and mature adipose tissue, centrally and peripherally (H&E, ×200) (b, c).

Microscopic examination showed the lesions with a thin fibrous capsule (Figure 2(a)) containing a large number of eosinophilic globules, elastic fibers, and collagen mixed tapes and mature adipose tissue, centrally and peripherally, in almost each high power field of the mass (Figures 2(b) and 2(c)).

Depending on these findings and the literature cases, the lesions were diagnosed as elastofibrolipoma.

## 3. Discussion

Elastofibroma is a benign lesion of ill-defined margins, encapsulated, which is often seen in the subscapular area in the elderly [3]. The lesion, which usually grows slowly, often causes back and shoulder pain [3, 4]. It can also be rarely located in various locations, such as the deltoid muscle, colon, and stomach [5, 6]. It has a central collagenous core with collagen fibers, elastic fibers and globules, and peripheral residual fat tissue [3, 7]. Elastofibrolipoma was first defined in 1995 by de Nictolis et al. in the anterior mediastinum and in 2005 by Erkiliç et al. in the subscapular area. de Nictolis et al. described the lesion, which was an encapsulated mass of central and peripheral adipose tissue mixed with elastic fibers and eosinophilic globules and fibrous bands, as elastofibrolipoma. de Nictolis et al. have classified this lesion as a lipoma variant of a true neoplasm. The subscapular lesion with similar characteristics, such as a thin fibrous capsule, with mature adipose tissue in each high power field and abnormal elastic fibers, which was defined by Erkiliç et al., was also diagnosed as elastofibrolipoma. Erkiliç et al. discussed whether this lesion should be classified as a lipoma variant or an elastofibrolipoma variant. In their study, they postulated that the lesion could be a lipoma variant due to its well-defined borders with a fibrous capsule and both central and peripheral adipose tissue, while the typical location in the subscapular area, the distribution pattern of the elastic fibers, and neoplastic properties of the adipose tissue could provide evidence for an elastofibroma variant. In our case, the excised larger tumoral mass localized in the left subscapular area, which is a typical location for an elastofibroma, had a thin fibrous capsule and was well-limited. Microscopic examination revealed abnormal elastic fibers and globules and mature adipose tissue mixed with collagen bands in all areas of the lesion. The radiological examination revealed another mass with a similar appearance, which was smaller and grew more slowly in the right subscapular area. In addition, histopathological examination of the smaller mass on the right side showed the diagnosis as bilateral elastofibrolipoma. Our case was diagnosed as elastofibrolipoma, based on the macroscopic, microscopic, and radiological findings. Because of the presence of the contralateral mass with similar radiographic and microscopic appearance we evaluated this case as bilateral. No bilateral elastofibrolipoma has been previously described. In the case of the presence of this lesion in the subscapular area, we recommend an investigation of the contralateral side, as well.

## 4. Conclusion

Depending on the findings such as thin fibrous capsule, containing a large number of eosinophilic globules, elastic fibers, and collagen mixed tapes and mature adipose tissue, centrally and peripherally, in almost each high power field of the mass the lesion was diagnosed as elastofibrolipoma. Because of the presence of the contralateral mass with similar radiographic and microscopic appearance we have evaluated this case as bilateral. Elastofibrolipoma is a rare entity and no bilateral elastofibrolipoma has been previously described.
